# Regulated dicing of *pre-mir-144* via reshaping of its terminal loop

**DOI:** 10.1093/nar/gkac568

**Published:** 2022-07-08

**Authors:** Renfu Shang, Dmitry A Kretov, Scott I Adamson, Thomas Treiber, Nora Treiber, Jeffrey Vedanayagam, Jeffrey H Chuang, Gunter Meister, Daniel Cifuentes, Eric C Lai

**Affiliations:** Developmental Biology Program, Sloan Kettering Institute, 1275 York Ave, Box 252, New York, NY 10065, USA; Department of Biochemistry, Boston University School of Medicine, Boston, MA 02118, USA; The Jackson Laboratory for Genomic Medicine, Farmington, CT 06032, USA; Regensburg Center for Biochemistry (RCB), Laboratory for RNA Biology, University of Regensburg, 93053 Regensburg, Germany; Regensburg Center for Biochemistry (RCB), Laboratory for RNA Biology, University of Regensburg, 93053 Regensburg, Germany; Developmental Biology Program, Sloan Kettering Institute, 1275 York Ave, Box 252, New York, NY 10065, USA; The Jackson Laboratory for Genomic Medicine, Farmington, CT 06032, USA; Regensburg Center for Biochemistry (RCB), Laboratory for RNA Biology, University of Regensburg, 93053 Regensburg, Germany; Department of Biochemistry, Boston University School of Medicine, Boston, MA 02118, USA; Developmental Biology Program, Sloan Kettering Institute, 1275 York Ave, Box 252, New York, NY 10065, USA

## Abstract

Although the route to generate microRNAs (miRNAs) is often depicted as a linear series of sequential and constitutive cleavages, we now appreciate multiple alternative pathways as well as diverse strategies to modulate their processing and function. Here, we identify an unusually profound regulatory role of conserved loop sequences in vertebrate *pre-mir-144*, which are essential for its cleavage by the Dicer RNase III enzyme in human and zebrafish models. Our data indicate that *pre-mir-144* dicing is positively regulated via its terminal loop, and involves the ILF3 complex (NF90 and its partner NF45/ILF2). We provide further evidence that this regulatory switch involves reshaping of the *pre-mir-144* apical loop into a structure that is appropriate for Dicer cleavage. In light of our recent findings that *mir-144* promotes the nuclear biogenesis of its neighbor *mir-451*, these data extend the complex hierarchy of nuclear and cytoplasmic regulatory events that can control the maturation of clustered miRNAs.

## INTRODUCTION

microRNAs (miRNAs) are an abundant family of ∼22 nucleotide (nt) RNAs, that mediate broad gene regulatory networks across diverse eukaryotic species, especially in plants and animals (1,2). In the canonical metazoan pathway, a primary miRNA (pri-miRNA) hairpin is first cleaved in the nucleus by the ‘Microprocessor’ complex, composed of the RNase III enzyme Drosha and its double-stranded RNA binding (dsRBD) partner DGCR8. This reaction releases the pre-miRNA hairpin, which is exported by Exportin-5 to the cytoplasm, where it is subsequently cleaved near its terminal loop by the RNase III enzyme Dicer to yield a small RNA duplex. This is loaded into an Argonaute effector, and matured to a single-stranded miRNA complex that seeks complementary targets for regulation. In addition, a variety of non-canonical miRNA substrates are known, including, a variety of pathways that can bypass Drosha, Dicer, or both, to yield functional miRNA species (3,4).

Although schematic pathways for miRNA biogenesis often imply a uniform and inexorable flow from precursor to effector complex, it is well-appreciated that all biological systems are subject to regulation. Over time, this has proven to be the case with the miRNA pathway as well, for which many different aspects are entry points for either positive or negative regulation that can collectively impact the efficacy and duration of the silencing reaction, on a global scale or on a miRNA-specific scale (5,6). A foundational concept for regulated miRNA biogenesis emerged with the control of let-7 maturation by Lin-28 family proteins. In this setting, sequence-specific recognition of *pre-let-7* loop by Lin-28 proteins inhibits let-7 maturation (7–11), owing to recruitment of terminal uridyltransferase (TUTase) enzymes that promote *pre-let-7* turnover (12–14). Based on this precedent, a number of other RNA binding proteins (RBPs) have been shown to associate with specific pri-miRNA or pre-miRNA targets to modulate miRNA biogenesis (5,6). Still, it is presumed that the full range of regulated miRNA biogenesis controlled by sequence-specific RBP interactions has only been partially elucidated, based on observations that (1) a substantial fraction of miRNA loci contain conserved loop sequences that imply constraint for RBP association (15), (2) largescale crosslinking-immunoprecipitation (CLIP) surveys indicate >100 RBPs interact with specific sets of pri-miRNAs in cells (16), and (3) largescale *in vitro* binding studies of miRNA precursors with cell lysates similarly recover dozens of relatively specific RBP:miRNA interactions (17).

Interleukin enhancer-binding factor 3 (ILF3) is one such factor that has been linked to control of miRNA biogenesis (16,18,19). In fact, ILF3 is documented as a multifunctional nucleic acid binding protein with highly diverse regulatory roles, and is referred to by multiple names in the literature. ILF3 generates isoforms termed nuclear factor 90/110 (NF90/110), which refer to the fact that NF90 and its heterodimeric partner NF45 (also known as ILF2) were originally identified from affinity purification of nuclear factors that interact with the NFAT binding site in the interleukin-2 promoter (20,21). NF90 was subsequently isolated as the M-phase phosphoprotein MPP4 (22) and NF110 was identified in the CCAAT box transcription factor complex as an RNA-binding component CBTF98/122 (23). One or both isoforms were isolated as translation control proteins TCP80/110 (24,25), as DRBP76, a dsRNA binding protein phosphorylated by interferon-inducible PKR (26), as NFAR1/2, nuclear factors that associate with dsRNA via the dsRNA-dependent kinase PKR (27). In this study, we generally utilize the locus name ILF3 (28) but refer to NF90 or NF110 during isoform-specific tests; we will also generally refer to their common heterodimeric partner as ILF2.

Overall, the biochemical functions and molecular pathways associated to ILF3 are myriad, and continue to expand (29). A shortlist of these activities reads like a who's who of fundamental gene regulatory processes, including but not limited to, transcriptional regulation (20,23,30), control of mitosis (31), repair of DNA breaks (32), miRNA biogenesis (33,34), splicing (35,36), ribosome maturation (37), regulation of circular RNA (38,39), transposon suppression (40), translational regulation (41,42), promotion of RNA stability (43), and inhibition of RNA editing (44). Consistent with such diverse functions, *ILF3* mouse knockouts are perinatal lethal (45) and transgenic *NF90/45* overexpression mice exhibit severe phenotypes including muscle atrophy (19). Nevertheless, it is challenging to rationalize how ILF3 obtains target specificity across all of these diverse regulatory transactions, and it is conceivable that some of the consequences of its modulation are due to a combination of direct and indirect effects. Thus, elucidation of how direct interactions of ILF3 with substrates might underlie regulatory effects, and perhaps obtaining separation of function proteins, is paramount when dealing with a highly multifunctional factor.

Relevant to this study is the fact that ILF3 has been connected to regulation of miRNA maturation (16,34). Again, the potential links within even this single pathway are myriad, and the consequences of ILF3 in miRNA production are inconsistent across the collected literature. NF90 associates with two core components of the canonical miRNA biogenesis pathway, the pre-miRNA export factor Exportin-5 (46) and the Drosha partner DGCR8 (47). While these associations might not necessarily be of functional consequence, the binding of NF90/45 complex was reported to inhibit nuclear processing of multiple primary miRNA transcripts in a transcription-independent manner, potentially by direct interaction with pri-miRNA transcripts (48). Of note, the latter study did not detect association of NF90/45 with either DGCR8 or Drosha, and suggested that the impact of NF90/45 on nuclear miRNA biogenesis was competitive with that of the Microprocessor (48). Reciprocally, transgenic expression of NF90/45 in mice led to suppression of many mature miRNAs and accumulation of their pri-miRNAs (19). Since both ILF3 isoforms contain two dsRBDs, it is plausible that NF90/110 proteins associate with double stranded portions of pri-miRNA hairpins. Subsequent studies broadened the concept that ILF3 associates with several dozen pri-miRNA loci (16), presumably interacting via double-stranded stem regions, and functions to suppress nuclear pri-miRNA cleavage (18,49,50).

In this study, we report that conserved sequences within the *mir-144* terminal loop play essential roles to gate its dicing. Mechanistically, our data indicate that *pre-mir-144* requires reshaping of the junction of the distal stem and the terminal loop into a base-paired form that is competent for *in vivo* dicing. We also provide evidence that the ILF3 complex (NF90/ILF2) positively regulates *pre-mir-144* dicing, in contrast to its reported role as an antagonist of miRNA biogenesis. Altogether, we demonstrate a critical post-transcriptional regulatory axis that governs miRNA maturation.

## MATERIALS AND METHODS

### Constructs

Plasmids for expression of all the miRNAs used in this study were constructed by inserting amplified DNA fragments containing the miRNA precursors from genomic DNA of HEK293T cells between Bgl II and Xho I sites downstream of a CMV promoter. The luciferase plasmids containing bulge or perfect miRNA sensors were constructed by inserting annealed DNA oligonucleotides containing miRNA sensor sequences between Nhe I and Xba I (for bulge sensors) or Xho I and Xba I (for perfect sensors) sites in the 3′ UTR of the firefly luciferase gene (84). The lentiviral shRNA plasmids were constructed by inserting annealed DNA oligonucleotides containing shRNA sequences between BamH I and Xba I site downstream of a U6 promoter. The cDNA plasmids for miRNA pathway factors were constructed by inserting amplified DNA fragments containing the ORF sequences from genomic DNA of HEK293T cells between Mlu I and Not I/Xba I sites downstream of a HA or Flag tag following a CMV promoter. All the details and oligonucleotide sequences used to clone these constructs are listed in Supplementary Table S1.

### Cell culture

HEK293T cells were grown in DME-high glucose media containing 10% FBS, 1% non-essential amino acids, 1% sodium pyruvate, penicillin/streptomycin, L-glutamate, and 0.1% 2-mercaptoethanol. K562 cells were grown in RPMI1640 media containing 10% FBS and 1% penicillin/streptomycin. Mycoplasma contaminations were regularly tested for the cell lines.

### Sensor assays

Transient transfection of the HEK293T cells with miRNA expressing plasmids (150–200 ng/well) and luciferase plasmids containing miRNA sensors (15 ng/well) was performed in 24-well cell culture plates using Lipofectamine2000 (Thermo Fisher) according to the manufacturer's protocol. Cells were harvested 24 h post-transfection and then Firefly and Renilla luciferase (co-transfected as reference gene) activities were measured using the Dual-Glo luciferase assay system (Promega).

### Knockdown assay using shRNAs

To knockdown ILF3 in K562 cells, we transduced cells with lentiviral shRNA constructs. **L**entiviral particles containing control or target shRNAs were produced by transfecting HEK293T cells with pMD2.G (400 ng/well), psPAX2 (800 ng/well) and the lentiviral shRNA plasmids (800 ng/well) using Lipofectamine 2000 (Thermo Fisher) in six-well plates. Cell culture supernatants were collected 48 hr after transfection and filtered through a 0.45 μm filtration membrane. K562 cells grown in six-well plates were then infected using lentiviral shRNA particles and selected by puromycin (4 μg/mL) for 4–5 days, and the surviving cells were collected for total RNA extraction.

To knockdown ILF3 in HEK293T cells, 2 μg of scramble or ILF3 targeting shRNAs were transiently transfected to HEK293T cells cultured in 6-well plates for 48 hr, then transfected with 400 ng of miRNA constructs. Cells were collected after 30 hr before extracting total RNA for analysis.

### Quantitative RT-PCR (qRT-PCR)

Total RNAs (1 μg) were extracted using Trizol, and used for cDNA preparation by DNase I treatment and reverse transcription using SuperScript III Reverse Transcriptase (Invitrogen). qPCR reactions were performed using SYBR Select master mix (Life Technologies). Data were normalized to *GAPDH* amplification. Three replicates were done for qPCR. Primer sequences for qPCR are listed in Supplementary Table S1.

### Co-immunoprecipitation assay

HEK293T cells grown in six-well plate were transiently co-transfected with plasmids (1 μg for each) encoding for HA-NF90 and Flag-tagged miRNA pathway factors or control gene. After 48 hr, the cells were washed with phosphate-buffered saline (PBS) and harvested in lysis buffer containing 50 mM Tris–HCl [pH 7.4], 150 mM NaCl, 1 mM EDTA, 1% Triton X-100 and protease inhibitor cocktail. After rotation at 4°C for 20 min, each lysate was clarified by centrifugation at 14 000 × g at 4°C for 15 min. A total of 500 μl supernatant was mixed with 10 μl of anti-HA magnetic beads (Thermo Fisher) at room temperature for 2 h. The beads were washed four times with Tris-buffered saline (TBS) and used for Western blotting analysis.

To test the RNA dependence of protein complexes, we added 100 U/ml RNase T1 and 40 μg/ml RNase A to the IP products after 2 h incubation, and placed them at 37°C for 20 min. Then the products were washed four times with Tris-buffered saline (TBS) before Western analysis.

### Northern blotting

To detect miRNAs from cultured cells, total RNA was prepared using Trizol reagent (Invitrogen). Equal amounts of total RNAs (10–15 μg) were denatured at 95°C and fractionated by electrophoresis on a 20% urea polyacrylamide gel. Then the gel was transferred to GeneScreen Plus membrane (Perkin Elmer), UV-crosslinked and baked at 80°C for 30 min and then hybridized with γ-^32^P-labeled probes at 42°C overnight. Probe sequences are listed in Supplementary Table S1.

To detect zebrafish miRNAs, a total of 20 injected embryos were collected at the indicated time points. Total RNA was extracted using Trizol (Invitrogen) and resuspended in formamide. Loading buffer 2× (8 M urea, 50 mM EDTA, 0.2 mg/ml bromophenol blue, 0.2 mg/ml xylene cyanol) was added and the samples were boiled for 5 min at 95°C. miRNAs were separated in 15% denaturing urea polyacrylamide gel in 1× TBE and then were transferred to a Zeta-Probe blotting membrane (Bio-Rad) using a semi-dry Trans-Blot SD (Bio-Rad) at 20 V (0.68A) for 35 min. Membranes were UV cross-linked and pre-hybridized with ExpressHyb Hybridization Solution (Clontech) for 1 h at 50°C. Membranes were blotted with 5′ ^32^P-radiolabeled DNA oligonucleotide probes at 30°C overnight. Membranes hybridized with oligonucleotide DNA probes where washed at room temperature with 2X SSC/0.1% SDS followed by 1× SSC/0.1% SDS for 15 min. The blots were exposed to a phosphorimaging screen for 1–3 days. Signal was detected using the Typhoon FLA 7000 phosphorimager (GE Healthcare Life technologies) and analyzed using the ImageQuant TL software (GE Healthcare).

### Western blotting

K562 cells were harvested and lysed for separation on 4–20% Mini-PROTEAN TGX Precast Protein Gels (Bio-Rad) and then transferred to a PVDF membrane. The blots were probed for 2 h at room temperature with rabbit polyclonal anti-ILF3 antibodies (Gunter Meister lab) diluted to 1:1000 or rabbit polyclonal anti-BUD13 antibodies (Bethyl Laboratories) diluted to 1:1000, or mouse monoclonal anti-β-tubulin antibodies (DSHB) diluted to 1:2000 and then incubated with a secondary antibody conjugated to horseradish peroxidase diluted to 1:5000. The signal was detected with Amersham ECL Prime Western Blotting Detection Reagent.

### *In vitro* Dicer cleavage assay

The pre-miRNAs were prepared from the microprocessing assay of the corresponding pri-miRNAs as described in our previous paper (51). The *in vitro* Dicer cleavage assays were done in a 30 μl reaction mixture containing 20 mM PIPES (pH 6.5), 1.5 mM MgCl_2_, 80 mM NaCl, 1 mM DTT, 10% glycerol, 1 U/μl of RNase inhibitor, 10 μl of IP-purified Flag-Dicer/TRBP complex and 2 μl of pre-miRNAs. Reactions were incubated at 37°C for 20 min. The reactions were stopped by adding 30 μl of 2× Gel Loading Buffer II. After heating at 95°C for 5 min, the samples were analyzed by Northern blotting.

### Purification of recombinant NF90 and NF45 proteins

Recombinant NF90 and NF45 protein fragments were expressed as N-terminal GST fusion proteins in *Escherichia coli* BL21 (DE3). The bacteria were lysed by sonication in PBS supplemented with 1 M NaCl and 2 mM DTT, and debris was removed by centrifugation. The protein was bound to glutathione-sepharose FF (GE healthcare) and eluted with PBS containing 10 mM glutathione. A subsequent gel filtration on a HiLoad S200 16/60 column was run in 50 mM HEPES pH 7.4, 200 mM NaCl, 2 mM DTT. The purified protein was mixed with 1 volume of glycerol and frozen at –80°C.

### Gel shift assay

Pre-miRNAs (1 pmol for each) were prepared as above from microprocessing assays and treated using CIP (NEB) in a 20 μl reaction system and then inactivated at 80°C for 2 min. The pre-miRNAs were then radioactively labeled as follows: 20 μl of de-phosphorylated RNA, 3 μl of 10x T4 PNK buffer, 3 μl of ^32^P-γ-ATP, 1 μl of T4 PNK (NEB) in a 30 μl reaction system and incubated at 37°C for 30 min. RNA were then purified by G-25 spin column (Fisher Scientific) and EDTA was added to 0.1 mM. RNA was heated to 95°C for 2 min and immediately chilled on ice. These oligos were incubated with different amounts (0, 0.5 or 2 ug) of recombinant NF90/45 proteins, 4 μl of 5× EMSA binding buffer (5×: 100 mM HEPES pH 7.9, 375 mM KCl, 2.5 mM DTT, 0.05% Tween 20, 50% glycerol), and water up to 20 μl. The binding reaction was incubated on ice for 30 min, followed by the addition of 2 μl of BlueJuice gel loading buffer (Invitrogen). Samples were then resolved on the pre-run 6% TBE retardation gel (ThermoFisher) at 100 V and then imaged.

### RNA Bind-N-Seq (RBNS)

Two 15 cm culture dishes of confluent HEK293 culture were harvested and lysed in 1 ml IP lysis buffer (50 mM Tris–HCl, pH 7.5, 300 mM KCl, 1 mM AEBFS, 1 mM DTT, 0.5% (v/v) NP-40). Insoluble material was pelleted by centrifugation (20 000 × g, 4°C, 15 min) and the supernatant transferred to a fresh reaction tube containing 20 μl Protein G Sepharose (GE Healthcare) prebound to anti-NF90/NF110 antibody from 20 μl rabbit antiserum. The binding reaction was incubated at 4°C for 2–3 h while agitating. The beads were then washed three times with 1 ml IP wash buffer (50 mM Tris–HCl, pH 7.5, 300 mM KCl, 0.05% (v/v) NP40). The immunoprecipitated proteins were used directly in an RNA-selection reaction by resuspending the beads in 400 μl binding buffer (25 mM Tris–Cl pH 7.5, 150 mM KCl, 3 mM MgCl_2_, 0.01% (v/v) NP-40, 1 mg/ml BSA, 1 mM DTT, 5% (v/v) glycerol, 15 μg/ml heparin and 0.1 U/μl Ribolock) and adding 15 μg of an RNA-pool with the sequence N_14_GUUU. The binding reaction was incubated for 30 minutes at room temperature while rotating.

The selected RNA molecules were sequentially ligated to a 3′ DNA adaptor (AAACTGGAATTCTCGGGTGCCAAGG-Amino-C7) and a 5′ RNA adaptor containing a T7 promoter sequence (GUUCAGUAAUACGACUCACUAUAGGG). The ligated product was reverse transcribed using the First Strand cDNA Synthesis Kit (Thermo Fisher) and the primer 5′-GCCTTGGCACCCGAGAATTCCAGTTT-3′. A PCR reaction was used to amplify the cDNA sequence and introduce barcodes for next generation sequencing (NGS).

To separate insert containing amplification products from empty adaptor sequences, the PCR reaction was run on a 6% urea PAGE gel and the band at 155 bp corresponding to the desired product was excised. The DNA was eluted overnight in 0.4 M NaCl and precipitated with ethanol. The redissolved PCR product was stored for NGS analysis.

To generate RNA for a second selection round, 50 ng of the PCR described above was amplified using the primers 5′-AATGATACGGCGACCACCGAGATCTACACGTTCAGTAATACGACTCACTATAGG-3′ and 5′-GCCTTGGCACCCGAGAATTCCAGTTT-3′. The resulting PCR product was purified using a PCR Clean-up Kit (Macherey Nagel) and cleaved by addition of 1.5 μl Fast digest MssI (Thermo Fisher), which recognizes the restriction site GTTTAAAC generated by the ligation of the RNA insert with the 3′ adaptor. The cleaved DNA was transcribed with T7 polymerase, which yields a new pool of RNAs with the sequence GGGN_14_GUUU. The RNA was purified by 18% urea PAGE, dephosphorylated with FastAP (Thermo Fisher) and monophosphorylated with polynucleotide kinase. 15 μg of the prepared RNA were used in a second selection cycle with freshly immunoprecipitated NF90.

Libraries from once and twice selected RNAs were sequenced on a MiSeq instrument (Illumina) with a 150 cycle MiSeq Reagent Kit to which we added a custom Read1 primer (5′-GATCTACACGTTCAGTAATACGACTCACTATAGGG-3′). Reads were barcode sorted and filtered for sequences containing the 3′ adaptor and the full MssI cleavage site, indicative of ligation of an intact RNA from the selection pool. After clipping of the adaptor and invariant sequences, only reads of the correct length (17 nt) were used for further analysis. The first three nucleotides were trimmed, and the resulting 14-mer sequences were analyzed for enriched sequence motifs using Streme (85) (v5.3.3) using the input RNA as a negative set with –rna –minw 6 parameters set.

### Zebrafish husbandry

Zebrafish were raised and maintained under standard fish facility conditions according to IACUC protocol #AN-5558 at Boston University. All injections were conducted in embryos from crosses of hybrid wild-type strains (AB/TU crossed to TL/NIHGRI).

### miRNA processing assay in zebrafish embryos

To generate different *pre-mir-144* hairpins universal oligo containing T7 promoter was annealed with specific oligo encoding indicated mutations in the *mir-144* stem loop and then filled by PCR. PCR products were purified using Monarch PCR&DNA Cleanup Kit (NEB) and used directly in *in vitro* transcription reaction performed overnight with AmpliScribe T7-Flash Transcription kit (Epicentre). *pre-mir-144* variants were purified from denaturing urea polyacrylamide gel. LNA-modified *pre-mir-144* hairpins were ordered from IDT. Pre-miRNA hairpins were injected into single-cell stage zebrafish embryos (1 nl of 10 μM stock) alone or together with 1 nL of 0.2 mg/ml alpha-amanitin (Sigma-Aldrich). Total RNA was extracted at 6 h after injection from 20 embryos using Trizol (Invitrogen) and their processing was analyzed by Northern blotting.

### microRNA activity reporter assay in zebrafish

Sequence encoding two imperfect miR-144 target sites (2× IPT-miR-144, 8-mers) was cloned into pCS2 + after coding sequence of EYFP. Reporter constructs were linearized with Not I restriction enzyme and *in vitro* transcribed with mMESSAGE mMACHINE SP6 Transcription Kit (Ambion). Zebrafish embryos were injected with 1 nL of 100 ng/μl of EYFP-*2xIPT-miR-144* reporter together with TagRFPT (86) as a control reporter. 1 nl of 10 μM each miR-144 hairpin was injected together with reporters. Embryos were imaged for EYFP and TagRFPT expression at 8 h after injection using a Zeiss Discovery microscope and photographed with a Zeiss Axiocam digital camera. Images were processed with ZEN software (Zeiss) and Photoshop CC19.16. EYFP and TagRFP-T fluorescence was quantified per embryo using ImageJ 1.52a.

### Public datasets

eCLIP bam files mapped to hg38 were downloaded from the encodeproject.org website (87). We used public databases to query expression of ILF3. Human BodyMap data: (https://www.ebi.ac.uk/gxa/genes/ENSG00000129351). Hematopoietic-specific data from Bloodspot: (http://servers.binf.ku.dk/bloodspot/?gene=ILF3).

## RESULTS

### The terminal loop of mammalian *pre-mir-144* is essential for its dicing

**Figure 1.**
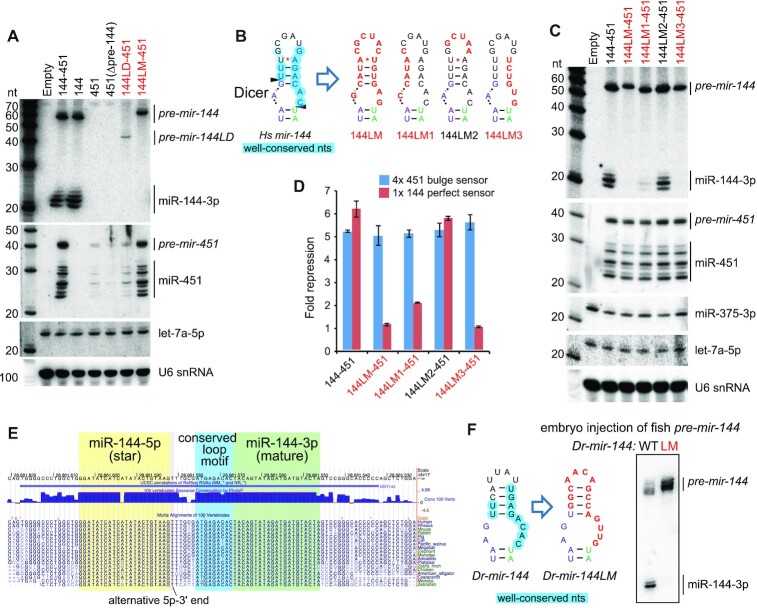
Processing of mir-144 requires its conserved terminal loop. (**A**) Transfection of human *mir-144/451* expression constructs into HEK293T cells and assayed by Northern blotting. We previously used these to show that biogenesis of *mir-451* requires Microprocessor enhancement via its neighbor *mir-144*. In particular, a variant deleted for the *mir-144* loop (*144LD-451*) is highly compromised for generation of both pre-miRNAs as well as both mature miRNAs. In contrast, specific mutations of the *mir-144* loop (*144LM-451*) support effective miR-451 biogenesis, but exhibit complete block as *pre-mir-144* hairpins. (**B**) Schematic of wildtype and loop variants of *mir-144*. The terminal loop nucleotides with high conservation are circled in blue on the wildtype structure (*Hs-mir-144*). In loop variants LM1-LM3, the nucleotides altered are in red. (C, D) Northern blotting (**C**) and luciferase activity assays (**D**) of the mutant *mir-144* constructs. Variants LM, LM1 and LM3 are defective in both assays, and according labeled in red. *mir-375* is used as transfection control for Northern blotting. (**E**) Alignment of the *mir-144* region across diverse vertebrate species illustrates the high degree of conservation of its terminal loop, especially the 3′ region adjacent to mature miR-144-3p. (**F**) Analysis of *Danio rerio* (*Dr*) *pre-mir-144* hairpins injected into 1-cell zebrafish embryos. Mutation of *Dr pre-mir-144* loop, while maintaining its structure, blocks its maturation. The terminal loop nucleotides with high conservation are circled in blue.

We recently characterized structure-function variants of the *mir-144/451* cluster (51), an operon composed of canonical *mir-144* and atypical (Dicer-independent, Ago2-dependent) *mir-451* (52). For simplicity, we transfected these constructs into HEK293T cells, which do not normally express erythroid-specific *mir-144/451*, and we adopted this strategy in Figure 1A. Notably, our prior efforts revealed that *mir-144* promotes the nuclear biogenesis of *mir-451*, which bears suboptimal features and requires a canonical neighbor to help recruit Microprocessor effectively (51,53). In contrast to the wild-type *144/451* operon, *mir-451* is not effectively matured when expressed as a solo hairpin or from an operon deleted for the *mir-144* hairpin (Figure 1A). In the course of these studies, we analyzed an operon bearing a deletion of the *mir-144* loop (*144LD-451*). The truncated *mir-144LD* hairpin has a very small terminal loop, similar to *mir-451*, and presumably recruits DGCR8 poorly. Accordingly, the accumulation *of pre-mir-144LD* is highly compromised relative to normal *pre-mir-144*, and *pre-mir-144LD* is incompetent at promoting the biogenesis of *mir-451* (Figure 1A).

Since the loop deletion disrupted *mir-144* biogenesis, we investigated the effect of mutating all nucleotides of the *mir-144* loop, while attempting to preserve its secondary structure (*144LM-451*). Surprisingly, this proved to be a separation-of-function mutant: miR-451 was matured normally, but miR-144 was fully blocked and instead accumulated as the *pre-mir-144* hairpin intermediate (Figure 1A). We interpret that *pri-144LM* recruits Microprocessor normally, and can promote miR-451 biogenesis, but that sequences and/or structures within the *mir-144* loop are subsequently essential for its cytoplasmic processing.

To gain further insights, we generated a series of loop variants (LM1/2/3) that alter specific aspects of the *mir-144* loop (Figure 1B). 144LM1 and 144LM3 change the left and right hand portions of the loop stem, respectively, while 144LM2 changes the identity of the apical unpaired loop. We find that LM3, which mutates the most conserved sequences of the *mir-144* loop, fully abrogates miR-144 maturation; 144LM1 was also strongly defective while 144LM2 was normal (Figure 1C). These data were mirrored by functional activity assays: a miR-451 luciferase sensor was similarly repressed by all *mir-144[LM#]/451* constructs, while a miR-144 luciferase sensor was similarly repressed by the wildtype operon and the 144LM2 variant (Figure 1D). By contrast, 144LM and LM3 were non-functional, while LM1 had modest activity.

We conclude that specific sequences in the *mir-144* loop are essential to promote its dicing, and are separable from the requirement of *mir-144* to enhance *mir-451* biogenesis. This requirement is striking given that (1) most miRNA regulatory paradigms are usually modulatory, rather than absolute, and (2) most regulators of Dicer cleavage repress, rather than promote, biogenesis (5,6).

### Conservation of loop-mediated dicing of *pre-mir-144* in fish

The *mir-144* loop is deeply conserved across vertebrates, with an extended region (∼9 nt) adjacent to the 5′ end of mature miR-144–3p exhibiting near identity across mammalian and avian species, and only minor changes in fish species within the very distal loop (Figure 1E, blue highlight). This highly constrained 3′ loop region engages in conserved base-pairing with the 5′ portion of the apical loop (Figure 1B). Accordingly, we used *Danio rerio* (*Dr*, zebrafish) to investigate whether the *mir-144* loop plays a substantial biogenesis regulatory role outside of mammals.

We synthesized *pre-mir-144* hairpins and injected them into one-cell stage zebrafish embryos, a setup that allowed us to isolate effects of the *mir-144* loop on Dicer-mediated processing from the preceding Microprocessor-mediated step. We compared a wildtype *Dr-pre-mir-144* with a loop mutant (LM) that changed its sequence but not overall structure (Figure 1F). Northern blotting showed that, as in human cells, wildtype *Dr-pre-mir-144* was efficiently converted into mature miRNAs whereas *Dr-144LM* was fully resistant to dicing (Figure 1F). The concordant behavior of human and fish *mir-144* strongly implicates that specific loop sequences are essential to turn *pre-mir-144* into a suitable Dicer substrate and that this regulatory step is independent of the preceding Drosha-cleavage event.

### Remodeling of the *pre-mir-144* terminal loop facilitates Dicer cleavage

**Figure 2.**
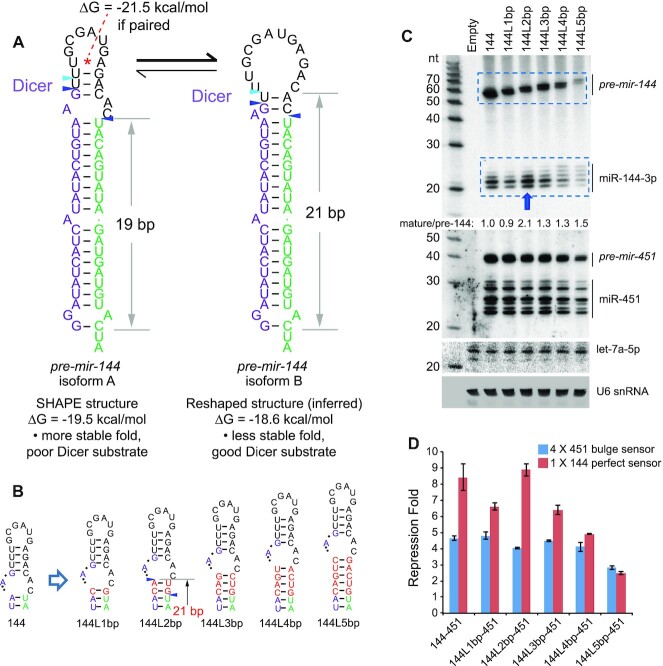
Evidence that *mir-144* is an intrinsically suboptimal Dicer substrate. (**A**) The structure on the left derives from experimental SHAPE-MaP profiling of *pre-mir-144*. The stem is short (19bp) and the Dicer cleavage site occurs in a local asymmetric bulge; both features are incompatible with effective processing. The asterisk designates a plausible basepair that was not confirmed in the SHAPE-MaP structure. The blue triangles represent dominant reads from small RNA sequencing; however, the aqua triangle is a well-expressed isomiR of miR-144-5p. The structure on the right is a predicted structure that conforms to known rules for effective Dicer substrates. Its stem length is 21bp, and the Dicer cleavage occurs 2 nts away from the junction of the duplex and the single-stranded loop. The aqua isomiR corresponds well to a 2-nt 3′ overhang from Dicer cleavage of this predicted structure. The free energies (Δ*G*) of *pre-mir-144* isoforms were predicted using *RNAstructure*. (**B**) Length variants in which 1–5 bp were inserted into the duplex stem just proximal to the bulge at the Dicer cleavage site. (**C**) The increasing stem length correlates well with decreasing accumulation of *pre-mir-144*, indicating compromised biogenesis. Nevertheless, insertion of two bp (144L2bp) resulted in increased mature miR-144-3p from the presumably lower level of hairpin precursor. By contrast, the changes in miR-144 biogenesis were largely segregated from those of miR-451. Effects were only seen with 144L5bp, whose substantially decreased *pre-mir-144* was associated with decreased *pre-mir-451* and miR-451, as expected from failure of Microprocessor enhancement on suboptimal *mir-451*. (**D**) Luciferase sensor assays confirm that 144L2bp maintains high activity, in contrast to all other length mutants that have decreased function. Activity of miR-451 was largely unaffected, except with 144L5bp.

To gain insight into the basis of *pre-mir-144* loop-mediated regulation we investigated the structure of *pre-mir-144* loop. Although *mir-144* appears as a typical canonical miRNA, as measured by its capacities to enhance *mir-451* biogenesis and to generate mature small RNAs efficiently (51), closer inspection revealed a non-canonical structural feature. Analysis of the inferred Dicer cleavage site, based on abundant small RNAs and the experimentally determined hairpin structure (51), coincides with a substantial asymmetric bulge positioned at the end of a 19 bp stem (Figure 2A). However, extensive structure-function studies of artificial shRNAs and variant miRNAs demonstrate that such a stem length and configuration are extremely unfavorable for effective and precise Dicer cleavage (54,55). Instead, a ‘loop-counting rule’ was proposed that an efficient Dicer cleavage site needs to be positioned 2nt from a bulge or loop within a stem of ∼21 bp (54). Inspection of *mir-144* revealed a potential alternate pairing of the apical stem that fulfills the loop-counting rule (Figure 2A) and is compatible with a 2-nt 3′ overhang on a substantial 3′ isomiR of miR-144-5p (Figure 1E). Of note, this preferred Dicer substrate structure is not intrinsically stable based on SHAPE-MaP profiling (51), which is also indicated by its higher free energy (Figure 2A).

We tested if *pre-mir-144* indeed presents a suboptimal Dicer substrate using *in vitro* processing assays. As a control, we used a remodeled Dicer-substrate *mir-451* variant (*mir-451LSM*) (51), which was efficiently diced to yield mature miR-451 (Supplementary Figure S1). By contrast, *pre-mir-144* resisted Dicer or Dicer/TRBP complexes *in vitro* (Supplementary Figure S1), suggesting the need of an additional regulatory factor.

To gain evidence that the atypical loop of *pre-mir-144* is responsible for its suboptimal dicing, we constructed a series of single basepair stem insertions just proximal to the internal bulge distal to the Dicer cleavage site, ranging from 1 to 5 bp (Figure 2B). Notably, all of these compromised the accumulation and function of mature miR-144, with one exception: the 2-bp insertion (*144L-2bp*) (Figure 2C, D). Given that this series of lengthening mutants was also associated with a linear decrease in the accumulation of *pre-mir-144* (Figure 2C), potentially reflecting deleterious effects of increasing stem length on nuclear biogenesis, the concomitant increase in mature miR-144 with *144L-2bp* was especially striking. In particular, the *144L-2bp* variant now fulfills the 2 nt loop-counting rule for effective Dicer substrates (Figure 2B). Consistent with these data, we observed that *144L-2bp* could now be diced *in vitro* (Supplementary Figure S1).

We note that these alterations to miR-144 biogenesis were largely decoupled from those of miR-451 in these operon constructs, whose biogenesis and activity were largely unaffected across these *mir-144* variants (Figure 2C, D). The exception was 144L5bp, which exhibits decreased accumulation of *pre-mir-144*. This reflects that its elongated stem structure is compromised as a Microprocessor substrate, and accordingly does not fully enhance production of *pre-mir-451*, and ultimately miR-451 (Figure 2C, D). This is consistent with recent reports on nuclear enhancement of *pre-mir-451* generation by a canonical miRNA neighbor (51,53), and reflects specificity of these assays. Overall, given that the primary sequence of *pre-mir-144* is highly conserved (Figure 1E), we infer that this locus specifically adopts features that prevent effective Dicer cleavage, which are overcome by some factor that recognizes its terminal loop. However, this regulatory layer can be bypassed in part with appropriate structural alterations.

### ILF3 positively regulates maturation of *pre-mir-144*

Are trans-acting factors involved in promoting *pre-mir-144* dicing? Prior studies (48) and recent ENCODE eCLIP profiling revealed ILF3 as an RBP that selectively regulates miRNA production (16). In K562 cells, which endogenously express *mir-144/451*, ILF3 was reported to associate with *mir-144* and act as a positive factor for its biogenesis (16). Its role here may be opposite than at other miRNA loci, since ILF3 was reported to inhibit miRNA biogenesis by sequestering dozens of pri-miRNAs from Microprocessor (18,48,50).

**Figure 3.**
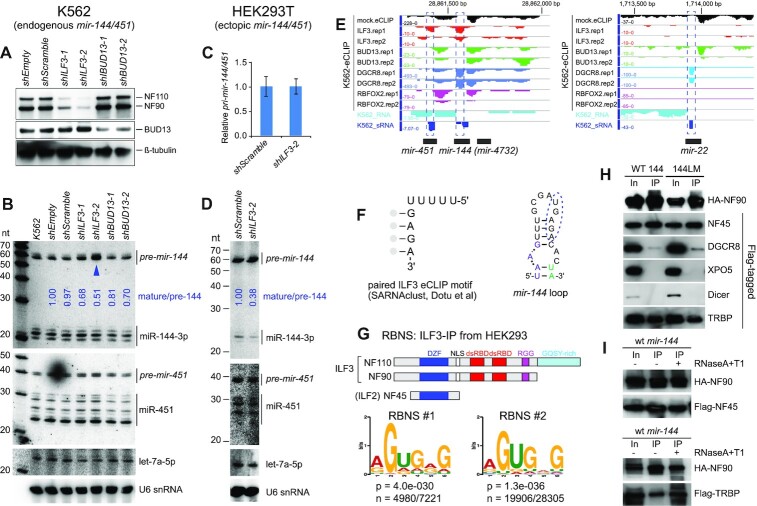
ILF3 is a positive regulator of *pre-mir-144* dicing. (A–D) Knockdown assays in K562 cells (A-B) and HEK293T cells (**C-D**). (**A**) Independent lentiviral shRNA constructs were transduced into K562 cells. Target proteins were evaluated by Western blotting after 5 days of knockdown. *shILF3-2* was slightly more effective than *shILF3-1*, while the two *shBUD13* constructs were comparable. (**B**) Northern blotting shows that only *shILF3* increased *pre-mir-144*, with a stronger effect in *shILF3-2* cells (blue arrowhead) that exhibit stronger ILF3 depletion. (**C**) Quantification of *pri-mir-144/451* transcripts following transfection in HEK293T cells. (**D**) Northern blotting of ectopic *mir-144/451* expression shows attenuated *pre-mir-144* dicing upon ILF3 depletion in HEK293T cells. (**E**) ENCODE K562 eCLIP data show that ILF3 and DGCR8 are selectively enriched at the *mir-144* terminal loop (*mir-22* shown for comparison). (**F**) ILF3 eCLIP data enriches a motif with sequence and structural similarity to the conserved motif in the *pre-mir-144* loop. (**G**) RNA Bind-N-Seq (RBNS) analysis using ILF3 (NF90/NF110) proteins immunoprecipitated from HEK293T cells. Independent RBNS datasets enriched for similar sequences, which resemble the eCLIP motif and the conserved *mir-144* loop. (**H**) ILF3 exhibits strong association to Dicer cofactor TRBP. Tagged constructs were co-expressed in HEK293T cells in the presence of *mir-144/451* (WT144) or a version bearing mutations in the loop nucleotides (144LM). HA-NF90 was immunoprecipitated (IP-ed), and bound proteins were compared between input (In) and IP samples. NF90 is a known heterodimeric partner of NF45 (ILF2), and this association was robust. NF90 was also reported to bind DGCR8 and Exportin-5 (XPO5); we validated modest association to the former but not the latter. Amongst other miRNA factors, the Dicer cofactor TRBP was strongly co-IPed with NF90. Additional co-IP data are shown in Supplementary Figure S3C. (**I**) RNase treatments show that both NF90-NF45 and NF90-TRBP complexes are independent of RNA.

Although the connection of ILF3 to miR-144 biogenesis was promising, we note a discrepancy. As mentioned, *mir-144* loop variants accumulate *pre-mir-144* but are blocked for miR-144 production (Figure 1), but *ILF3* knockdown was reported to strongly deplete *pre-mir-144* (by 7-fold), as well as mildly reduce its mature products (16). We re-examined this by generating independent lentiviral shRNA constructs for *ILF3* and *BUD13*, which were both reported as direct regulators of *mir-144* (16). When transduced into K562 cells, *ILF3*-knockdown depleted both NF90 and NF110 protein isoforms, while *BUD1*3-knockdown suppressed its cognate protein product (Figure 3A). Of note, as *ILF3* suppression eventually induced lethality in K562 cells, we collected cells after 4–5 days of shRNA induction. At this timepoint, *shILF3-*2 induced stronger loss of NF90/110 proteins than *shILF3-1* (Figure 3A).

Northern blotting of K562 knockdown cells showed modest changes in mature miRNAs, with only mild reduction in miR-144-3p upon BUD13 suppression (Figure 3B). It is likely that the documented high stability of mature miRNAs (56) compromised our ability to observe alterations in their levels during these transient experiments. The knockdown timecourse was necessarily limited by cell lethality of ILF3 depletion, and the cells accumulate high levels of endogenous miR-144 before the knockdown was initiated (Figure 3B, K562 lane, as well as other control shRNA-treated cells). Nevertheless, we observed substantial increases in *pre-mir-144* in cells with ILF3 loss, with reproducibly higher elevation in *shILF3-2* cells (Figure 3B, arrowhead), which exhibit greater depletion of ILF3 proteins. These data resolve that ILF3 is in fact well-positioned to serve as the positive regulator of *pre-mir-144* dicing inferred from our mutational studies. However, other potential roles of ILF3 on maturation of miR-144 remained possible.

To better understand the effects of ILF3 on *mir-144* processing, we depleted ILF3 in HEK293T cells (Supplementary Figure S2A) and then transfected *mir-144/451* construct. Since HEK293T cells do not express this miRNA cluster endogenously (Figure 1A), this setup permits us to assay dependence of newly-transcribed *mir-144/451* on endogenous ILF3. qPCR tests did not show change of *pri-mir-144/451* transcripts upon *ILF3* knockdown (Figure 3C). However, Northern blotting showed that accumulation of mature miR-144 was selectively attenuated upon ILF3 depletion, relative to miR-451 or let-7a (Figure 3D). These data support that ILF3 promotes dicing of *pre-mir-144*, but not microprocessing of *pri-mir-144*.

ILF3 generates two isoforms, NF90 and the C-terminally extended variant NF110 that includes a GQSY-rich domain; both of these heterodimerize with the NF45 subunit (Supplementary Figure S2B). We conducted additional tests to evaluate the contributions of distinct factors. We were able to efficiently suppress the accumulation of NF110 using independent shRNAs (Supplementary Figure S2C). In addition, ILF2 (NF45) stabilizes its heterodimeric partners, and one of the NF45 shRNAs induced substantial reduction in NF90/NF110 proteins, in accordance with previous observations (57). Using these constructs, we find that effective depletion of ILF2 also led to substantial accumulation of *pre-mir-144*, while loss of NF110 had only mild effects (Supplementary Figure S2D). However, there may be compensation between ILF3 isoforms, since depletion of NF110 led to increased NF90 (Supplementary Figure S2C). This extends the previous observation of extensive cross-regulatory interactions amongst ILF2 and ILF3 proteins (57). We also document specificity in the effects on *pre-mir-144* accumulation, since ILF3-knockdown did not substantially affect *pre-let-7a* or *pre-mir-21* (Supplementary Figure S2E).

We conclude that heterodimers of ILF3 isoforms with ILF2 specifically promote miR-144 biogenesis at the dicing step. However, as NF90 exists at higher levels in K562 cells, and is largely able to promote *mir-144* biogenesis in the absence of NF110, the distinct protein isoform encoded by NF110 may not be critical in this process.

### Evidence that ILF3 may recognize the *pre-mir-144* loop

Prior studies used *in vitro* binding assays to provide evidence that ILF3 associates with several pri-miRNAs (18,48–50). However, the intrinsic double stranded RNA binding activity of ILF3 makes it challenging to interpret results of this assay. In particular, while ILF3 was shown to associate modestly with *pri-mir-144 in vitro*, another locus lacking evidence of eCLIP binding or regulation (*pri-mir-20a*) showed even stronger apparent association with ILF3 *in vitro* (16). Thus, despite the apparently specific enrichment of ILF3 at the *pre-mir-144* loop compared to other expressed miRNAs in eCLIP data (Figure 3E), it has been difficult to infer specific *in vivo* miRNA targets of ILF3 from *in vitro* binding assays. We tested this using gel-shift assays, and observed that recombinant NF90 formed gel-shift complexes with *pre-mir-144* that were super-shifted upon addition of ILF2 (Supplementary Figure S3A). However, NF90 also formed effective complexes with control *pre-let-7* (Supplementary Figure S3A) as well as loop-mutated *pre-mir-144LM* that is not regulated by ILF3 (Supplementary Figure S3B). These data emphasize prior concerns that in vitro binding assays of ILF3 with pre-miRNA may not reflect specific interactions (16). Moreover, the apparent challenge in assembling a sequence-specific complex may explain why we were not able to recapitulate stimulation of NF90/ILF2-mediated dicing *in vitro* (Supplementary Figure S1).

Our recent analysis of ENCODE ILF3 eCLIP data revealed enrichment of a UUUUUGAGA motif in ILF3 peaks, in both a linear format and a paired format where the GAGA was preferentially located within a stem (58). This was striking, since the regulatory region of the *mir-144* loop bears a highly conserved UGAGA, where the 3′ sequence resides within a stem (Figure 3F). To follow on this, we used RNA Bind-N-Seq (RBNS) (59) to analyze the site preferences of ILF3 proteins. For this purpose, we carried out experiments using *in vivo* ILF3 proteins (using antibodies that immunoprecipitate both NF90/NF110, likely in complex with ILF2/NF45). Interestingly, replicate RBNS experiments enriched for similar motifs (Figure 3G and Supplementary Figure S4), which bear homology to the regulatory sequence in the *pre-mir-144* loop and the eCLIP motif.

Taken together, even though ILF3 proteins recognize dsRNA relatively non-specifically via their dsRBDs, these data also suggest that ILF3 complexes may be involved in sequence-specific recognition of *mir-144*.

### ILF3 interacts with miRNA biogenesis factors

Our data begin to outline a model in which the ILF3 complex may remodel the *pre-mir-144* terminal loop to permit its dicing. We wondered if such a mechanism might involve direct connections with the miRNA biogenesis machinery. Although their names reflect that their original isolation as ‘nuclear factors’ bearing nuclear localization signals, NF90, NF110 and NF45 (ILF2) are shuttling proteins that exist in the cytoplasm (57) and have documented cytosolic regulatory functions (34). Therefore, even though NF90 was previously reported to form complexes with XPO5 (46) and DGCR8 (47), we sought to perform more comprehensive tests across the miRNA pathway. We also performed these co-IP assays in the presence of ectopic *mir-144* or *mir-144LM*, in case any interactions might be potentiated by excess miRNA substrate.

As a control, we found that NF90 exhibited robust interactions with its heterodimeric partner NF45 (Figure 3H). We could recapitulate published interactions of NF90 with DGCR8 but these were modest; the heterodimeric partner of DGCR8, Drosha, was not co-IPed with NF90. We also observed minor interactions of NF90 with Ago2, consistent with prior largescale profiling (60), but none with XPO5, Ran, Dicer, or negative control luciferase (Supplementary Figure S3C). Interestingly, the strongest interactions between NF90 and any miRNA pathway component involved the cytoplasmic Dicer dsRBD partner TRBP (61) (Figure 3H). None of these interactions were substantially different in the presence of wildtype versus mutant *mir-144*. Moreover, NF90 complexes with NF45 and TRBP survived treatment with RNase A + T1, demonstrating that these robust interactions are not only independent of specific target substrates, but independent of RNA in general (Figure 3I).

Based on these data, we tested if supplementing *in vitro* dicing reactions with recombinant NF90/ILF2 proteins could improve miR-144 biogenesis, with or without TRBP. However, none of the conditions tested rescued maturation of miR-144 (Supplementary Figure S1). It is possible that an appropriate multiprotein regulatory complex is not formed under the conditions we tested. Alternatively, NF90/45 might have sequestered *pre-mir-144 in vitro*, thereby preventing dicing. Similar to other reports (18,48,50), we observed in gel-shift assays that NF90 protein can bind *pre-mir-144*. However, we show that *in vitro*, NF90/NF110 can bind several pre-miRNAs that are presumably not targeted by endogenous ILF3 (Supplementary Figure S3A, B). This mirrors other reports of likely non-specific interactions of ILF3 with pre-miRNA hairpins *in vitro* (16). Therefore, it may be challenging to design appropriate *in vitro* conditions that can reconstitute the hypothesized ILF3-mediated dicing of *pre-mir-144*.

### Reshaping of the *pre-mir-144* terminal loop determines its biogenesis

Since *in vitro* reconstitution was not possible in our hands, we performed additional experiments to support our model for regulated maturation of miR-144. We assayed a finer set of sequence alterations designed to alter sequence-specific contacts of the inferred ILF3 binding site, alter the secondary structure, or to disrupt the capacity of apical loop remodeling. We also tested chemical modifications of normal *pre-mir-144* bases designed to impair loop reshaping. These tests support the following interpretations regarding *pre-mir-144* processing.

**Figure 4.**
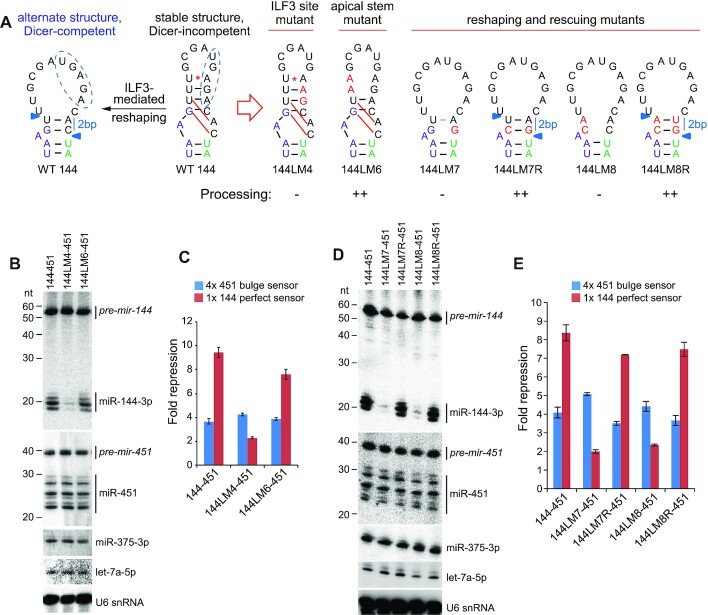
Reshaping of the apical loop mediates dicing of *pre-mir-144*. (**A**) Schematic of wildtype and variant *mir-144* loop constructs. In the wildtype *mir-144* loop (WT 144), the stable SHAPE-MaP structure is indicated as a Dicer-incompetent state. Nucleotides predicted to re-pair as a Dicer-competent substrate (with 2bp stem from the single-stranded junction to the Dicer cut site) are connected by red lines; the putative ILF3 binding site is indicated with a blue dashed oval. In the *mir-144* variants, the mutated nucleotides are in red. (**B**) Northern blotting and (**C**) activity sensor assays highlight the ‘GA’ core of the ILF3 site is essential for miR-144 processing and function (144LM4). However, the apical stem structure is not required (144LM6). (**D**) Northern blotting and (**E**) activity sensor assays of reshaping mutants show that if the 2 bp re-structured stem cannot form, the resulting *pre-mir-144* variants remain Dicer-incompetent (144LM7 and 144LM8). By contrast, restoration of pairing in the reshaping mutants (144LM7R and 144LM8R) fully restored *pre-mir-144* dicing.

1. *Importance of the inferred ILF3 binding site*. We made finer mutations that disrupt the conserved loop motif (Figure 4A) that bears similarity to the ILF3/NF90 RBNS-selected site (Figure 3G), while preserving the stem structure they reside in. Alteration of two nts in the 3′ region (144LM4) nearly abolished miR-144 biogenesis (Figure 4B). These effects were mirrored by miR-144 activity assays (Figure 4C), while none of these mutants substantially affected miR-451. In particular, the conserved GA dinucleotide, which is enriched in ILF3 eCLIP data and RBNS data, is critical for miR-144 biogenesis.
2. *Role of reshaping within the terminal loop*. Our model proposes that sequences outside of the presumed ILF3 binding site, in particular ones involved in alternate basepairing configurations, also mediate miR-144 biogenesis. We tested the involvement of the pairing context of the presumed ILF3 binding site. Our initial structure-function tests hinted that disruption of overall 5′ apical stem sequence, which alters stem structure, was deleterious to *mir-144* maturation (Figure 1C). One of these mutants (144LM1) maintained the full ILF3 binding site. However, a more targeted disruption of the 5′ apical stem (144LM6) exhibited fairly normal miR-144 biogenesis (Figure 4B). In light of our subsequent data, we re-interpret this to suggest that the primary effect of 144LM1 may not be on the structured ILF3 binding site, but instead on the capacity of this hairpin to adopt an alternative Dicer-compatible structure (Figure 2A).
  To test this notion, we introduced a C-to-G mutation in position adjacent to the 5′ end of miR-144–3p (144LM7); i.e. at the Dicer cleavage site (Figure 4A). Strikingly, even though this cytosine is neither involved in base-pairing in the experimentally determined secondary structure (Figure 2A), nor part of the inferred ILF3 binding site (Figure 3G), this single change strongly impaired miR-144–3p biogenesis and function (Figure 4D, E). We then mutated two nts on the 5′ side of the apical loop that we inferred to mediate reshaping (144LM8) and found that these were similarly defective for miR-144 biogenesis (Figure 4D, E). Nevertheless, all of the mutants supported normal maturation of miR-451. Furthermore, we constructed additional variants that restore the reshaping base pairs in 144LM7 and 144LM8 (144LM7R and 144LM8R, respectively, Figure 4A) and found that these fully rescued dicing of corresponding *pre-mir-144* mutants (Figure 4D, E).

Altogether, these tests provide strong support to the notion that re-pairing within the apical stem is critical for *pre-mir-144* dicing, and emphasize that these regulatory events are fully separable from the impact that *pri-mir-144* has to enhance *mir-451* processing.

### Loop reshaping is required for *pre-mir-144* dicing in fish

**Figure 5.**
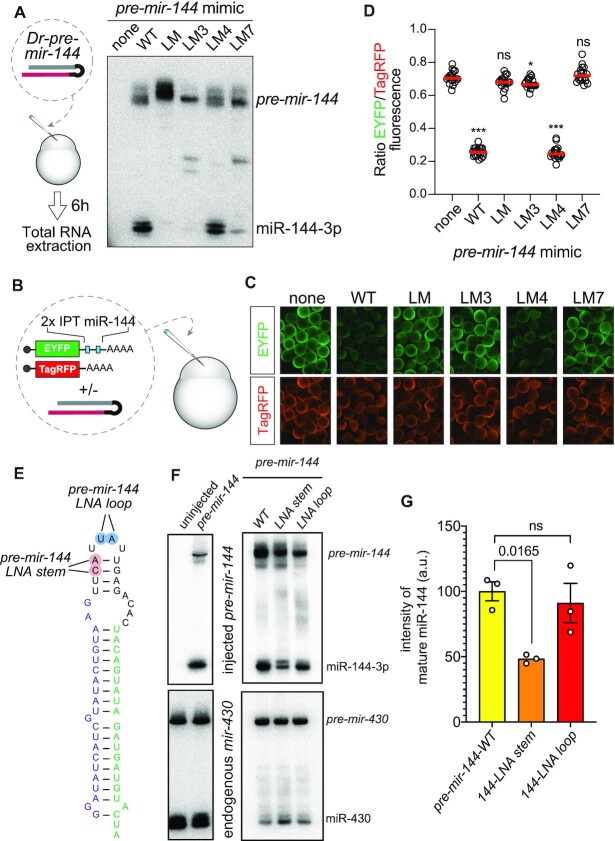
Loop-mediated biogenesis of zebrafish miR-144. (**A**) Scheme for analysis of wildtype and mutant *Dr-pre-mir-144* hairpins after injection into 1-cell zebrafish embryos. Mutations of the entire loop (LM), the 3′ loop sequence (LM3), or of a single bulged nucleotide inferred to mediate apical loop reshaping (LM7) block conversion of *pre-mir-144* into miR-144-3p. (**B**) Activity assay of *pre-mir-144* variants. (**C**) Loop mutant versions of *pre-mir-144* are defective in repression of a miR-144 sensor. (**D**) Quantification of the reporter assays. (E–G) Reinforcement of the stable base-pairing in the preferred *pre-mir-144* loop secondary structure impairs maturation of miR-144. (**E**) Variants of zebrafish *pre-mir-144* bearing pairs of LNA substitutions, but otherwise retain wildtype sequence. The ‘LNA stem’ variant affects re-shaping of the hairpin structure, but the ‘LNA loop’ variant is predicted to be neutral. (**F**) Northern blot showing processing of injected *pre-mir-144* molecules, showing selective impairment of mature miR-144 from the LNA stem mutant compared to wildtype and LNA loop variant. (**G**) Quantification of mature miR-144 levels from independent injection and Northern assays, using one-way ANOVA test.

Since we have shown that the *mir-144* loop is essential for its maturation of miR-144-3p in fish (Figure 1F), we exploited this system to test conservation of reshaping principles. As before, we injected precursor hairpins into one-cell stage embryos and analyzed their maturation using Northern blotting. For these assays, we generated additional synthetic *Dr-pre-mir-144* variants with the equivalent changes as in human variants (Supplementary Figure S5). These tests indicate that LM4 was matured at the same level as wild-type *Dr-pre-mir-144*, suggesting less of a specific-sequence requirement as in mammals. However, maturation of point mutants LM3 and LM7 was nearly completely abrogated, similar to the complete loop mutant LM (Figure 5A). We corroborated this with functional activities, by co-injecting *pre-mir-144* variants with fluorescent sensors for miR-144-3p and a non-cognate control (Figure 5B). Qualitative imaging and quantitative analysis confirm that LM, LM3 and LM7 mutants were specifically defective for target regulation (Figure 5C, D). These results indicate that zebrafish miR-144 is processed only when the loop structure is conducive to adopt alternative structures.

The fish assays are advantageous in that they directly assay maturation of *pre-mir-144* molecules. Thus, these tests demonstrate that structure-dependent regulation of *pre-mir-144* is independent of both its normal context within the *mir-144/451* cluster, as well as potential regulation during pri-miRNA processing. As a final test of the model of structural remodeling, we analyzed variant *pre-mir-144* molecules bearing pairs of LNA substitutions (Figure 5E). When introduced into the apical stem (144-LNA stem), LNA modifications increase the pairing energy and are predicted to favor the non-productive fold of *pre-mir-144*. To control for unanticipated effects of LNA substitutions, we tested a control variant bearing two LNA within the unstructured terminal loop (144-LNA loop), which we showed is not required for regulated miR-144 biogenesis (Figure 1B–D). Importantly, both LNA variant molecules retain wildtype sequence. When injected into zebrafish embryos, we observed that production of mature miR-144-3p was reduced for the LNA-stem modified precursor, whereas the LNA-loop mutant exhibited similar biogenesis as unmodified wild-type *pre-mir-144* (Figure 5F). The results of independent replicate injection and Northern analyses are quantified in Figure 5G. These results reinforce our notion that *pre-mir-144* must adopt an alternative, unfavorable, structure in order to be cleaved by Dicer, and that double-stranded capacities of the *pre-mir-144* loop play a central role in the process of remodeling.

### The ‘loop-counting rule’ accounts for defective dicing of *pre-mir-144* variants

**Figure 6.**
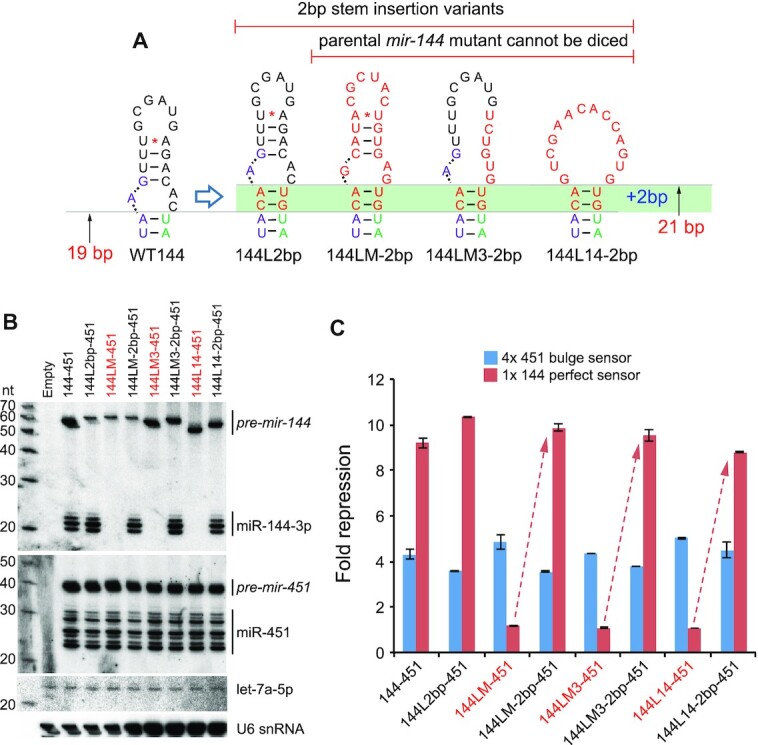
Structural remodeling of the apical stem can fully rescue defective *mir-144* variants. (**A**) Schematics of 2bp insertion variants of *mir-144* mutants. The wildtype mir-144 loop exhibits a 19 bp stem to the Dicer cleavage site on the 3p arm (Figure 2A). As shown in Figure 2, insertion of a 2bp stem just distal to the Dicer cleavage site, rendering a 21 bp stem, impairs production of *pre-mir-144*, but results in a relative enhancement of mature miR-144-3p yield. The green bar indicates similar 2 bp stem insertions into other *mir-144* mutants that are incompetent for Dicer cleavage. (**B**) Northern blotting of the panel of unmodified *mir-144* mutants (labeled red) and their companion 2 bp insertion variants shows rescue of mature miR-144 production. (**C**) Luciferase sensor assays shows rescue of miR-144-3p activity by 2bp insertions in all defective *mir-144* variants.

If the role of ILF3 is to counteract the intrinsically suboptimal structure at the *pre-mir-144* cleavage site, then we might be able to bypass its requirement by making appropriate structural changes. In particular, our finding that a 2bp insertion just distal to the Dicer cleavage site can enhance miR-144 biogenesis, even though this otherwise appears to render it a suboptimal miRNA substrate (Figure 2), provided the rationale for rescue assays. We therefore inserted the 2bp stem into three *mir-144* variants that were largely incapable of producing mature miR-144-3p. We tested these *mir-144/451* backbones from Figure 1B: 144LM, with all loop nucleotides mutated but preserving overall apical stem structure; 144LM3, with mutations in the 3′ region of the terminal loop including the inferred ILF3 site; and 144L14, where the 17 nt *mir-144* terminal loop was exchanged for a 14 nt sequence lacking overt structure (Figure 6A). All of these variants were incompetent for miR-144–3p maturation, but insertion of the 2 bp stem provided full rescue (Figure 6B). Note that while the biogenesis of mature miR-144–3p was fully activated in all 2bp insertions, there was correspondingly little effect on maturation of miR-451 from these operon constructs. This was consistent with the notion that effects on *mir-144* biogenesis occurred after cleavage by Microprocessor. We observed similar, specific, rescues when assaying the activity of miR-144-3p and miR-451 using sensor assays (Figure 6C).

**Figure 7.**
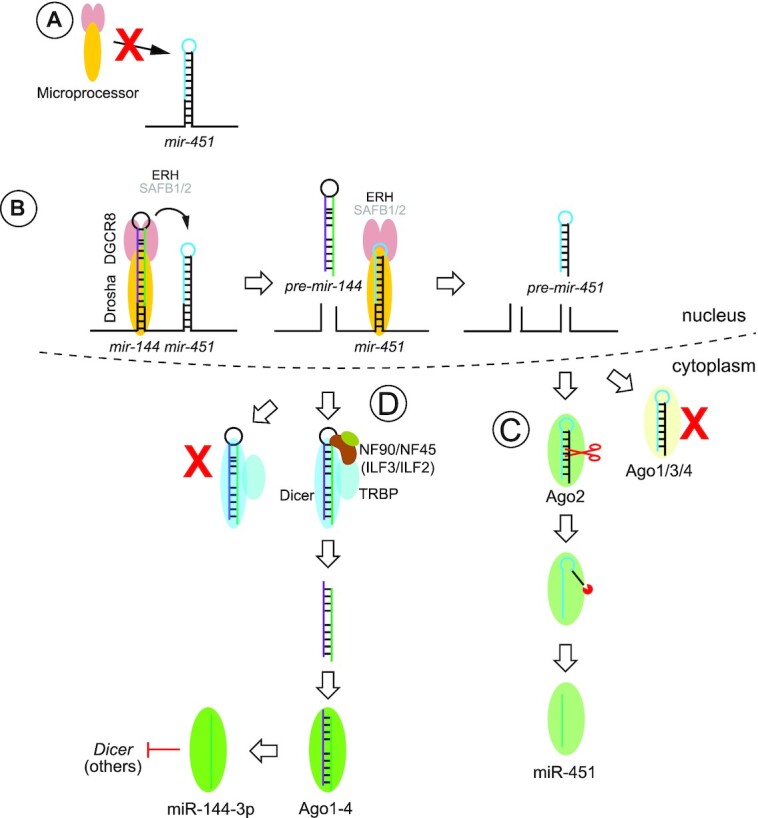
Summary of complex regulatory interactions that govern biogenesis of the *mir-144/451* cluster. (**A**) *mir-451* has a very short stem and small terminal loop, rendering it a suboptimal substrate of the nuclear Microprocessor complex (Drosha + 2DGCR8). (**B**) Instead, it requires assistance from its operon neighbor *mir-144* for effective recruitment and/or cleavage by Microprocessor. Efficient nuclear *mir-451* biogenesis requires the accessory factor ERH. The requirement of SAFB proteins, recently implicated in suboptimal miRNA biogenesis at clusters, is unknown. (**C**) The short hairpin of *mir-451* escapes Dicer cleavage but can load directly into Ago effector proteins. Association with non-slicing effectors (Ago1/3/4) is a dead-end path. If *pre-mir-451* associates with Ago2, it can be cleaved on the 3′ arm and then further resected by PARN nuclease to yield mature miR-451. (**D**) *pre-mir-144* presents a suboptimal Dicer substrate, on account of its slightly short stem and location of Dicer cleavage site within an asymmetric internal loop. Its terminal loop contains a recognition sequence for ILF3 (NF90/NF110) in complex with ILF2 (NF45), which can recruit the Dicer cofactor TRBP. We hypothesize the ILF3 complex remodels the apical stem region into a form that is competent for Dicer cleavage. In the erythroid lineage, one of the targets of mature miR-144-3p is Dicer itself, which is proposed to lead to downregulation of canonical miRNAs and permit upregulation of Dicer-independent miR-451.

Taken together, these variant assays provide support to our model for regulated processing of *pre-mir-144*, involving reshaping of its suboptimal apical hairpin stem into a form that supports Dicer cleavage (Figure 7).

## DISCUSSION

### Regulation of miRNA biogenesis via terminal loops of hairpin precursors

The regulation of selected *pre-let-7* hairpins by binding of Lin28 proteins via their terminal loops (7–11) established a paradigm for RBP-mediated control of miRNA biogenesis, whereby its zinc knuckle domains and cold shock domain interact with specific loop motifs in cognate targets within the let-7 family hairpins (62,63). Since many other miRNAs exhibit conserved terminal loop sequences, this implies that a constellation of other potential regulatory interactions that control miRNA biogenesis (15). However, relatively few of these have been characterized in detail, or are known to have very strong effects on target miRNA biogenesis.

Amongst other cases of locus and/or sequence-specific RBP regulators studied, many more affect the nuclear (Drosha) step than regulate the cytoplasmic (Dicer) step (5). Since Microprocessor serves as the gatekeeper for canonical miRNA biogenesis, it may make sense for this processing step to have acquired diverse locus-specific regulation that suits the functional activities and requirements of the miRNAs in question. A number of RBPs have been described to bind within the terminal loop to positively regulate Drosha cleavage of target miRNAs (e.g. hnRNPA1, KSRP, SMAD, TDP-43, RBFOX, SRSF1), while the binding of others inhibits Drosha cleavage (e.g. hnRNPA1, LIN28B, HuR/MSI2, RBFOX, YB-1) (5,6). Depending on the context, the same RBP has been described to promote or inhibit miRNA biogenesis. With regard to modulation of Dicer cleavage, few cases have emerged beyond Lin28 RBPs, and these examples are not as well understood. TDP-43 promotes Drosha and Dicer cleavage of select miRNAs by binding terminal loops, but mechanisms that underlie its target specificity or that promote either RNase III cleavage are unclear (64). YB-1 was reported to inhibit both Drosha and Dicer cleavage of *mir-29b-2* by binding to its terminal loop via a cognate site, and potentially by occluding access to these miRNA ribonucleases (65).

Our studies reveal a critical regulation for Dicer regulation of *pre-mir-144* involving ILF3. Amongst miRNA regulators proposed to do more than simply shield recognition by Drosha and/or Dicer, certain precedents are relevant for the inferred action of ILF3. In the case of *pri-mir-18a*, binding of hnRNPA1 was proposed to stimulate Drosha cleavage by reshaping its stem loop structure (66,67). In addition, Lin28 binding was shown to remodel the terminal loop of *pre-let-7*, yielding a conformation that is inhibitory to Dicer cleavage while concomitantly promoting 3′ modification by TUTases (68). Interestingly, Lin28a was also shown to exert positive and negative influence on processing of different miRNAs (69), which mirrors the observation of dual function of ILF3 on miRNA biogenesis from our and previous studies. We hypothesize that ILF3 directly recognizes *pre-mir-144* via a cognate binding site, and that its action remodels its terminal loop into a form that is amenable to Dicer cleavage; this may also be facilitated by the recruitment of TRBP by ILF3. However, we acknowledge a limitation of our study that we were not able to reconstitute the dicing enhancement *in vitro*. Therefore, an alternative possibility is that the ILF3 complex helps recruit another factor that mediates reshaping of *pre-mir-144*. In any case, we provide strong evidence that the conserved structural layout of the junction between duplex and the apical loop in *pre-mir-144* constrains its dicing potential, in a manner that conforms to established rules that describe suboptimal Dicer substrates (54). Notably, not only can we abolish *pre-mir-144* processing with specific mutations within its loop, but we can also bypass this regulatory control via appropriate structural alterations.

### Dual modes of target interaction for ILF3/NF90: structural and sequence-specific

ILF3 proteins (NF90 and NF110) have been studied in diverse regulatory contexts, including both transcriptional and many distinct post-transcriptional regulatory processes. Based on their dual dsRBD architecture, ILF3 proteins might be presumed to interact with target RNAs on mostly structural grounds. For example, endogenous circular RNAs were reported to generally adopt limited amounts of double stranded character and thus generically bind dsRBD proteins including NF90/NF110 (38) and PKR (70). Prior RIP-seq analysis of NF90 and NF110 in hESCs revealed overlapping and distinct targets, but no specific binding motifs (57). Standard RIP strategies may not provide sufficient resolution to infer binding site enrichment, although an earlier RIP-chip study suggested that several NF90 targets contained AU-rich motifs (41).

Recently, generation of largescale RBP eCLIP data (71) permitted the inference of *mir-144* as an NF90 target (16). Moreover, analysis of the NF90 eCLIP data revealed enrichments for specific types of nucleotide motifs, one of which closely resembles the highly-conserved apical loop-stem sequence that we characterized as essential for *pre-mir-144* dicing (58). RBNS data in this study supports that NF90/NF110 have selectivity for an overlapping site (bearing a UGAG core). However, it remains to be seen if ILF3 proteins, with or without partner ILF2 (also known as NF45), associate specifically with the *pre-mir-144* loop. Our studies indicate that it is challenging to identify *in vitro* conditions that reveal stable and specific association of NF90/NF45 to wildtype *pre-mir-144*, relative to mutant *pre-mir-144* or other pre-miRNAs, possibly due to their intrinsic affinity to dsRNA. Nevertheless, the structural analysis of the NF90/NF45 heterodimer (72) and the tandem dsRBDs of NF90 on RNA reveal the capacity for base-specific interactions with guanine and adenine in the minor groove (73). Such observations set the precedent that it is conceivable that ILF3 proteins may recognize both structural and sequence features in their targets. Alternatively, they may associate generically with targets via structural elements, but these may be augmented by the presence of preferred sequence features.

### Multiple regulatory interactions govern the biogenesis of the non-canonical *mir-144/451* cluster

While the conserved vertebrate *mir-144/451* operon was identified over 15 years ago (74), the many intricacies of its biogenesis have been slow to emerge. Nevertheless, each of these may inform broader principles of miRNA biogenesis and utility (Figure 7).

The first bombshell was the discovery that *mir-451* encodes a Dicer-independent locus that is instead fully dependent on Ago2 catalysis for maturation and thus function (75–77). Although *mir-451* appears to be the only well-conserved miRNA that utilizes this strategy, the Ago2-dependent strategy is highly adaptable for direct reprogramming to produce arbitrary silencing RNAs (75,78), and can also be grafted into synthetic biogenesis strategies that may offer advantages (4,55).

Because the short stem and small terminal loop of *mir-451* impairs its capacity for effective recruitment and/or processing by Drosha/DGCR8 (Microprocessor), its neighbor *mir-144* plays a sequence-independent, proximity-dependent, role to promote nuclear cleavage of *mir-451* (51,53). Although *mir-451* is particularly dependent on *mir-144*, owing to its atypical biogenesis mechanism, the strategy of neighbor assistance of suboptimal nuclear miRNA processing is more broadly applicable (51,53,79).

In this study, we introduce an additional regulatory layer, in that *mir-144* is a rare miRNA subject with an essential requirement for activation of its cytoplasmic biogenesis. This contrasts with many other miRNA regulatory paradigms, where characterized trans-acting RBPs often induce only quantitative and/or minor alterations to miRNA biogenesis. Although the full effect is difficult to visualize with endogenous manipulations, owing to requirement of ILF3 for viability in K562 cells and the long life of pre-existing miRNAs, we show that even point mutations in its binding site or in the proposed site of structural remodeling can fully block conversion of its pre-miRNA into mature miRNA. Such a strong requirement for trans-acting factors to facilitate canonical miRNA biogenesis is unexpected, given the history of flexible reprogramming of miRNA backbones for gene silencing purposes, and the fact that miRNAs do not collectively share obligate features for accessory motifs that point to accessory factors (80,81). Notably, the recognition of these regulatory strategies permits the decoupling of these idiosyncratic regulatory mechanisms; i.e. the role of *mir-144* to promote miR-451 can be substituted by other miRNAs or by appropriate mutations within the *mir-451* hairpin. Here, we further show that *mir-144* mutants that are fully incompetent for cytoplasmic maturation can nevertheless efficiently promote miR-451 biogenesis, while reciprocally, we can bypass the need for structural remodeling of *pre-mir-144* by introducing alterations that convert it into a conventional Dicer substrate.

Because all of these idiosyncrasies of *mir-144/451* appear to be well-conserved across vertebrates, we imagine that they evolved to optimize the spatial expression, the temporal accumulation and/or the levels of these miRNAs. *mir-144/451* are highly expressed in the erythroid setting. Another Ago2-dependent locus, the Dicer-dependent hairpin *mir-486*, is also highly upregulated in the same setting (82). This suggests that red blood cell precursors have uniquely evolved to exploit Ago2-dependent miRNA biogenesis strategies, concomitant with downregulation of non-catalytic Ago paralogs (82). However, even within *mir-144/451*, the levels of the two mature miRNAs are discrepant, with miR-451 becoming far more abundant. This led to proposal of a negative feedback loop for interconnected miRNA biogenesis pathways in blood, whereby miR-144 directly targets Dicer, which eventually compromises canonical miRNA biogenesis and enhances maturation of Dicer-independent miR-451 (83).

Here, we elucidate an additional unexpected step for *mir-144* regulation in that conserved sequences in the *pre-mir-144* loop are involved in alternative structures, and that productive Dicing involves reshaping the apical stem into an extended duplex (Figure 7). We also provide evidence that ILF3 proteins (NF90 and perhaps NF110), in complex with their heterodimeric partner ILF2 (NF45), are involved in this process. Although ILF3 is broadly expressed, its expression is modulated. Of note, ILF3 transcripts rise from the common myeloid progenitors to megakaryocyte-erythroid progenitors (MEPs), where *mir-144/451* begin to be expressed, and remain higher in early erythroblasts than in more committed cells of the erythroid lineage (Supplementary Figure S6). Thus, it is conceivable that decreasing ILF3 expression during erythroid differentiation, alongside other mechanisms described above, helps to skew the maturation of miRNAs from an individual operon.

## Supplementary Material

gkac568_Supplemental_Files
