# Supplementary material for: Regulated dicing of pre-mir-144 via reshaping of its terminal loop
Source: Nucleic Acids Res. 2022 Jul 8;50(13):7637–54. doi: 10.1093/nar/gkac568 (PMC9303283; doi:10.1093/nar/gkac568)
Supplement: gkac568_Supplemental_Files [file gkac568_supplemental_files.zip › 2022_0501_ILF3_mir144_supp_merge_print.pdf]

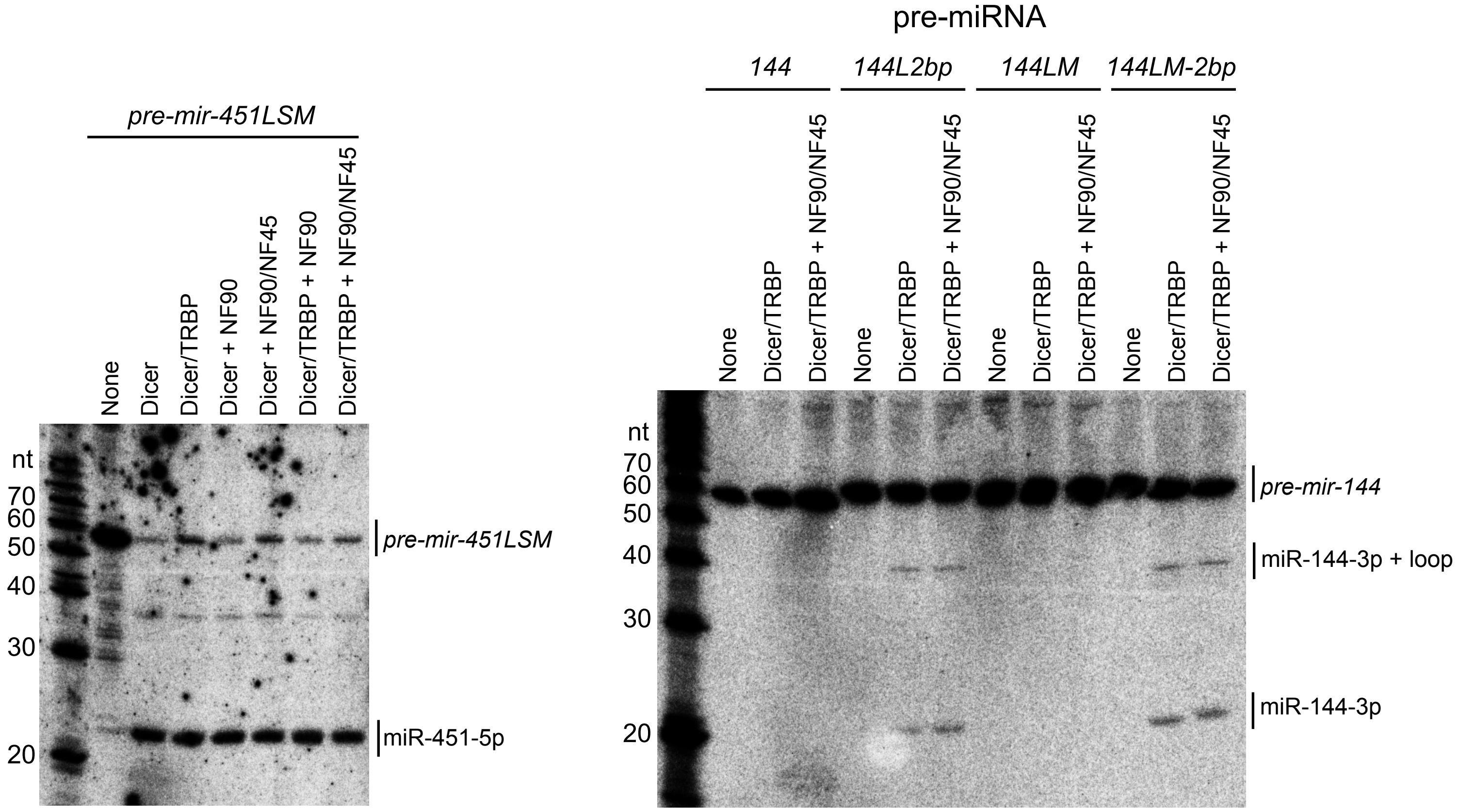

Supplementary Figure 1. *In vitro* evidence that *pre-mir-144* is a poor Dicer substrate.

(Left) *pre-mir-451LSM* is a reprogrammed version of *mir-451* that is Dicer-dependent. Upon incubation of *pre-mir-451LSM* RNA with immunoprecipitated Dicer or Dicer/TRBP complex, or Dicer complexes supplemented with bacterially expressed NF90 and/or NF45 proteins, mature miR-451-5p is effectively produced. (Right). *pre-mir-144* is the wildtype hairpin, and *pre-mir-144-LM* contains loop mutations; the corresponding L2bp variants contain 2bp insertions into the apical stem that bypass inferred requirement for ILF3-mediated loop remodeling (see Figure 6). Neither *pre-mir-144* nor *pre-mir-144-LM* are effectively Diced *in vitro*, even when supplemented with NF90/NF45, but their 2bp insertion mutants can be Diced.

Shang et al,
Supplementary Figure 1

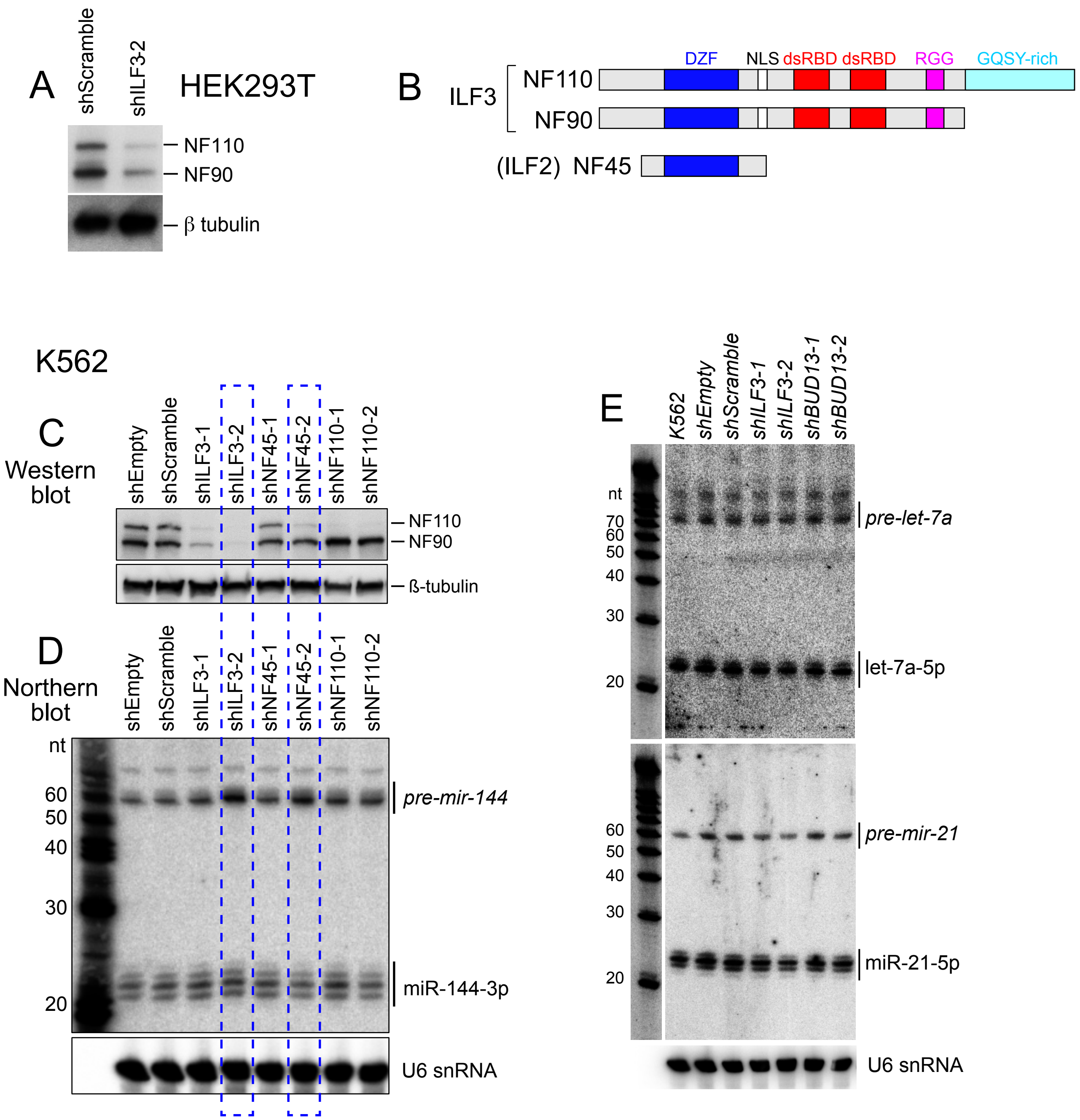

Supplementary Figure 2. Additional characterization of ILF3 and NF45 requirements for miR-144 biogenesis.

(A) Western blot validation of shRNA-mediated knockdown of ILF3 (NF90/NF110 protein isoforms) in HEK293 cells (relevant to main Figure 3C-D). (B) Domain structure of ILF3 isoforms (NF90 and NF110) and their heterodimeric partner NF45 (also known as ILF2). (C) Western blot validation of shRNA-mediated knockdown of the indicated factors in K562 cells (relevant to main Figure 3A-B). (D) Northern blotting using miR-144-3p probe. Accumulation of *pre-mir-144* hairpin is observed upon shILF3-2, which yielded stronger knockdown of NF90/110 proteins than shILF3-1, as well as upon knockdown of shNF45-2, which reduced NF90/110 proteins. Comparably mild effects on *pre-mir-144* accumulation were observed upon shNF110 knockdown, but we note that this was associated with upregulation of NF90 protein. (E) Knockdown of ILF3 or BUD13 did not appreciably affect hairpin precursors for *pre-let-7a* or *pre-mir-21*.

Shang et al,
Supplementary Figure 2

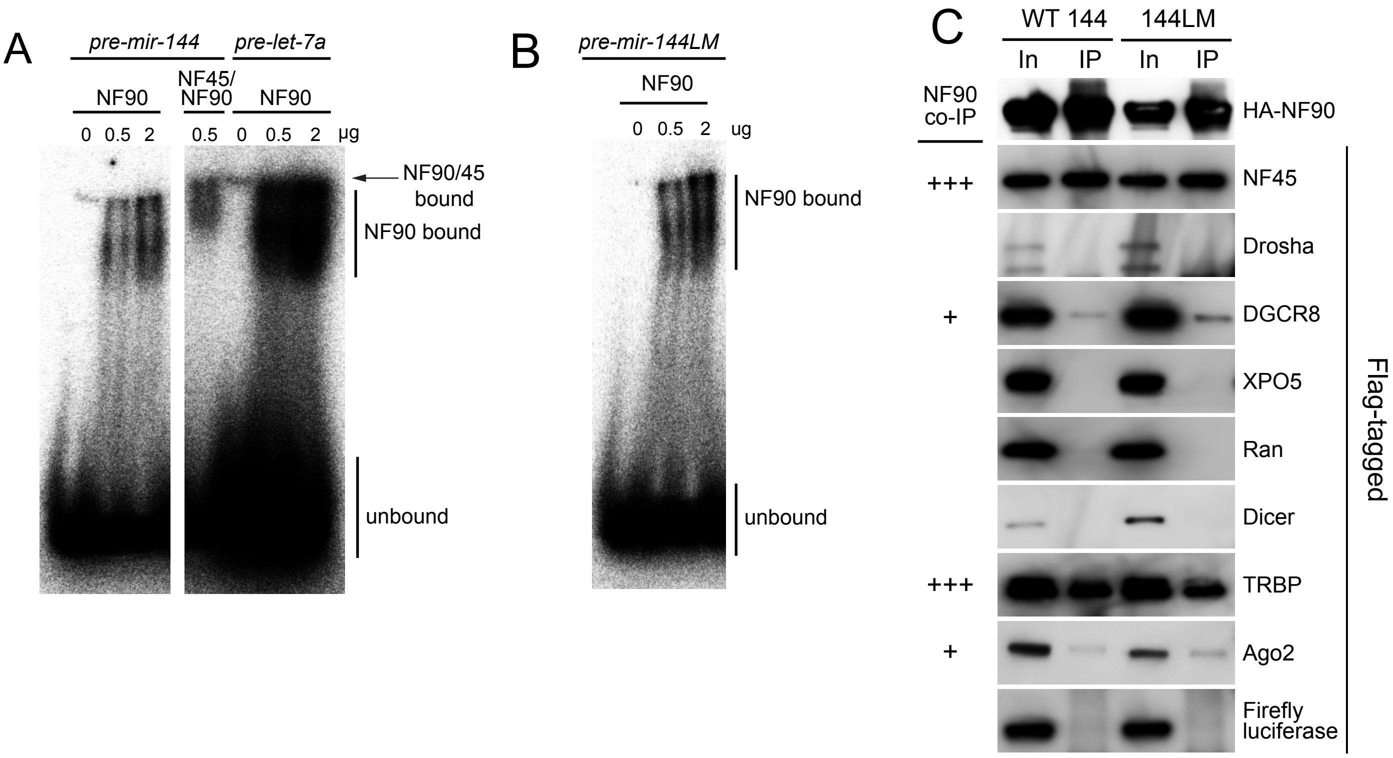

Supplementary Figure 3. Evidence for NF90 pre-miRNA binding and protein complexes.

(A) Gel shift assays using radiolabeled pre-miRNAs and bacterially expressed NF90 or NF90/45 proteins. NF90 associates with *pre-mir-144* and is super-shifted by NF45; however, NF90 also associates with *pre-let-7a*. (B) NF90 also binds with mutant *pre-mir-144LM*, which is defective for in vivo processing (Figure 1). Thus, in vitro binding of pre-miRNAs by NF90 complexes seems to be non-specific. (C) Systematic assessment of protein partners of NF90 using co-immunoprecipitation (co-IP) assays. HF-NF90 was tested for association with various Flag-tagged components of the miRNA pathway. NF90 associates well with its known partner NF45, but barely bound reported partners DGCR8 and Ago2, and did not co-IP with reported partner XPO5. Instead, we recovered TRBP as a novel strong partner of NF90. A subset of these NF90 co-IP data are also shown in main Figure 3I.

Shang et al,
Supplementary Figure 3

NF90/110 endogenous 14mer RBNS #1

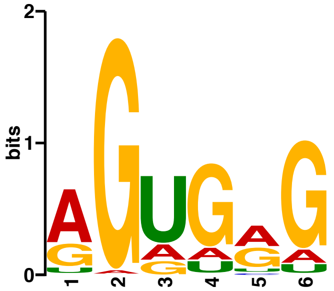

P-value	sites
4.0e-030	4980/7221
5.3e-005	248/7221

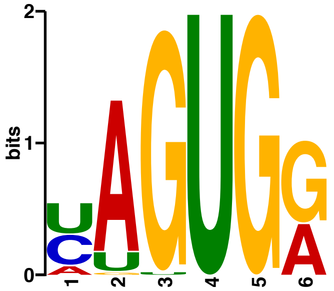

NF90/110 endogenous 14mer RBNS #2

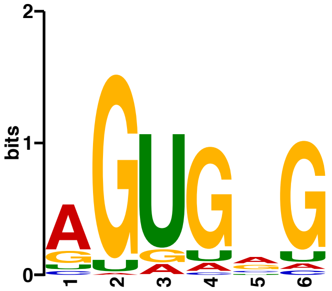

P-value	sites
1.3e-036	19906/28305
2.1e-004	1490/28305

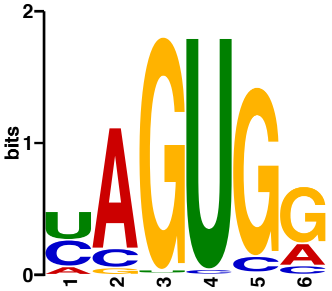

Supplementary Figure 4. All significant ILF3 (NF90/NF110) RBNS motifs.

Two independent RNA Bind ‘n’ Seq experiments were performed using a randomized 14 mer pool and endogenous materials immunoprecipitated with ILF3 antibodies. The antibodies do not distinguish NF90/NF110 isoforms, which are expected to be complexed with ILF2 (NF45). We used Streme to identify enriched motifs relative to input. These were the only hits recovered in both RBNS libraries.

Shang et al,
Supplementary Figure 4

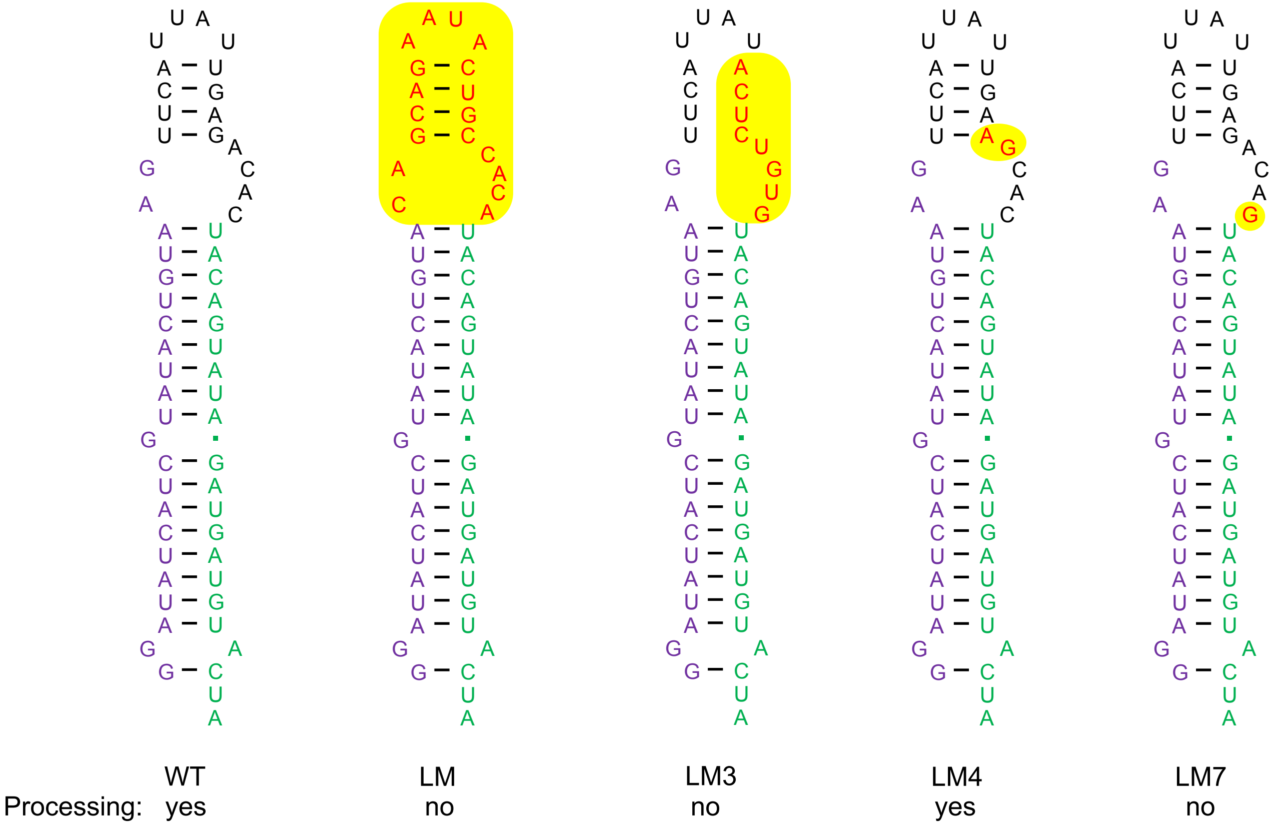

Dre-pre-mir-144 variants

WT: GGAUAUCAUCGUAUACUGUAAGUUCAUUAUUGAGACACUACAGUAUAGAUGAUGUACUA
LM: GGAUAUCAUCGUAUACUGUACAGCAGAAUACUGCCACAUACAGUAUAGAUGAUGUACUA
LM3: GGAUAUCAUCGUAUACUGUAAGUUCAUUAUACUCUGUGUACAGUAUAGAUGAUGUACUA
LM4: GGAUAUCAUCGUAUACUGUAAGUUCAUUAUUGAAGCACUACAGUAUAGAUGAUGUACUA
LM7: GGAUAUCAUCGUAUACUGUAAGUUCAUUAUUGAGACAGUACAGUAUAGAUGAUGUACUA

Supplementary Figure 5. Structures and sequences of zebrafish *pre-mir-144* variants tested.

Relative to wildtype (WT) *Dre-pre-mir-144*, the loop mutant (LM) versions bear changes marked in red that disrupt the sequence and/or structure of the terminal loop. LM changes all loop nucleotides but preserves its structure; LM3 mutates the 3' portion of the terminal loop; LM4 alters two nucleotides in the putative ILF3 site; LM7 changes a single bulge nucleotide that is inferred to mediate reshaping of the apical terminal loop.

Shang et al,
Supplementary Figure 5

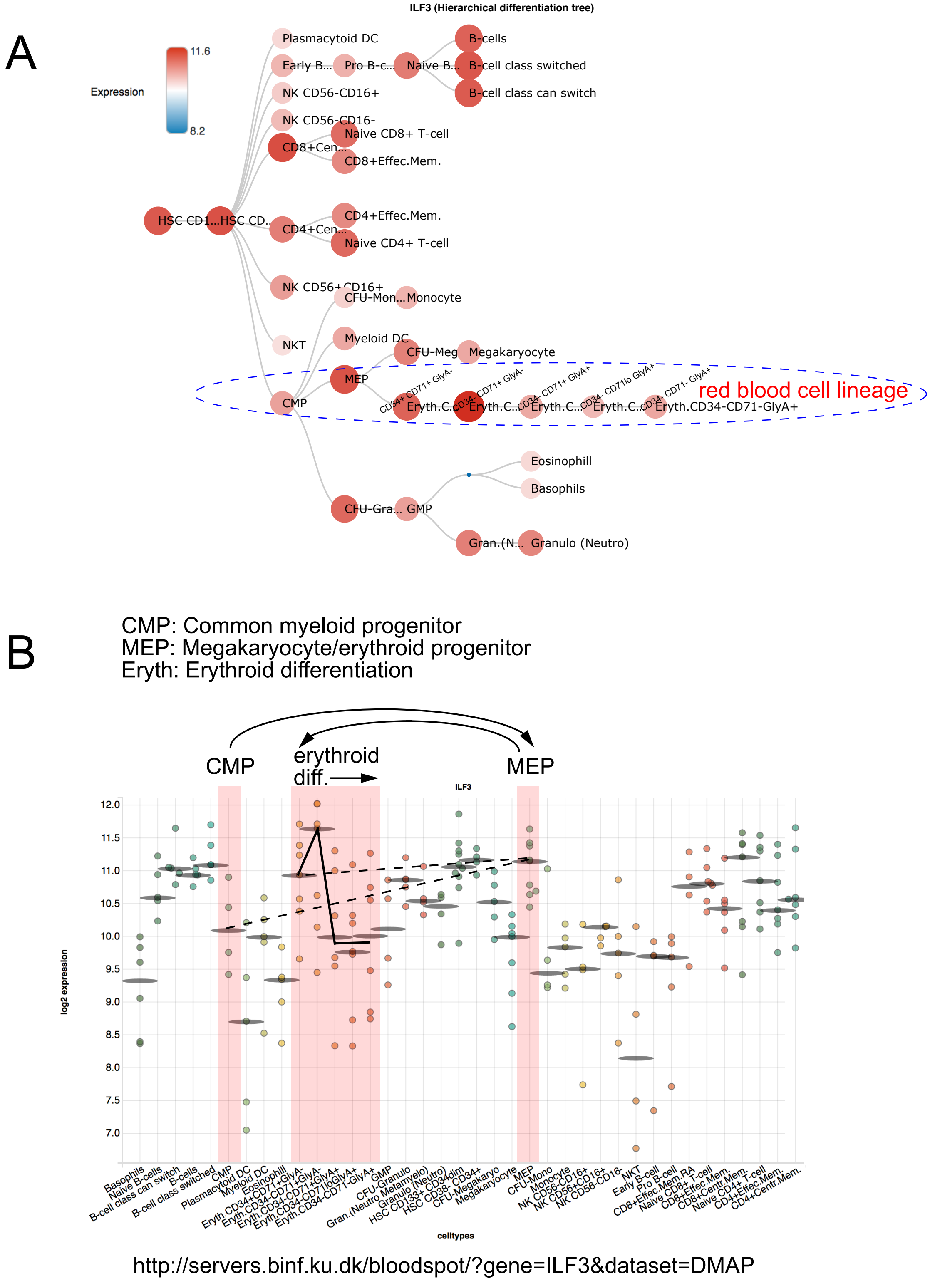

Supplementary Figure 6. Dynamic expression of ILF3 in the hematopoietic compartment and erythroid lineage.

(A) Expression of ILF3 in hematopoietic lineages, as summarized from BloodSpot. The blue dotted oval emphasizes the red blood cell lineage. Note that ILF3 increases from CMP to MEP stages, coinciding with the increase in miR-144 maturation, and is maintained in early committed erythoblasts but declines as they differentiate along the red blood cell lineage. (B) Details of ILF3 gene expression, with the red blood cell lineage highlighted.

Shang et al,
Supplementary Figure 6
